# Supplementary material for: Influence of Job Insecurity on Musculoskeletal Disorders: A Mediation Model with Nursing Aides
Source: Int J Environ Res Public Health. 2023 Jan 15;20(2):1586. doi: 10.3390/ijerph20021586 (PMC9865540; doi:10.3390/ijerph20021586)
Supplement: Supplementary file 1 [file ijerph-20-01586-s001.zip › ijerph-2158246-supplementary.pdf]

Table S1. Results regarding workers´demographic data

Demographic data	Percentage (%) or Mean (SD)
**BMI (kg/m²)**	
M(S)	25.52 (4.76)
**Time at current job (months)**	
M(S)	168.1 (105)
**Weekly working hours**	
M(S)	35.62 (2.51)
**Civil status**	
Married	54.8
Single	22.9
Separated	22.3
**Education level**	
Lower secundary	0.6
Upper secundary	11
Vocational training	74.4
University	12.7
Master or above	1.3
**Work shift**	
Fixed work	3.3
Shift work	93.5
Split Shift	0.6
Irregular	2.7
**Smoke**	
Non-Smoker	53.7
Ex-smoker	17.5
Smoker	28.8
**Alcohol**	
No	53.3
Eventually	46.3
On a daily basis	0.4
**Physical activity**	
No	13.9
Less than once a month	10.1
More than once a month	76

BMI: body mass index; M: mean; SD: standard deviation
